# Prevalence of *Toxoplasma gondii* antibodies among waste collectors in Kuwait

**DOI:** 10.1016/j.parepi.2025.e00431

**Published:** 2025-05-29

**Authors:** Anfal Yousef

**Affiliations:** Department of Medical Laboratory Sciences, College of Allied Health Sciences, Kuwait University, Kuwait

**Keywords:** *Toxoplasma gondii*, Toxoplasmosis, One health, Kuwait, Parasite, Parasitic disease, Protozoa

## Abstract

**Background:**

Toxoplasmosis is a parasitic disease with a global burden, particularly affecting pregnant women, neonates and immunosuppressed individuals. Latent toxoplasmosis has also been associated with neuropsychological disorders in immunocompetent individuals. In Kuwait, the unregulated presence of stray cats in residential areas may contribute to toxoplasmosis spread within the community. The targeted population of this study were waste cleaners, a potentially higher risk group in the community. The aim of the study was to estimate the seroprevalence of *Toxoplasma gondii* among waste collectors across two governorates of Kuwait – Kuwait City and Jahra.

**Method:**

Blood samples were collected from 201 waste workers from two different cities: 53 and 148 workers from Kuwait City Governorate and Jahra Governorate, respectively. These samples were analysed to determine the seroprevalence of *T. gondii* antibodies (IgM and IgG) and IgG avidity using a chemiluminescence immunoassay.

**Results:**

The findings revealed that a total of 1 % and 21 % of waste workers had *T. gondii* IgM and IgG antibodies in their sera, respectively. Furthermore, 26 % of seropositive workers exhibited high avidity, indicating that infections were likely not recent. Almost half of seropositive workers (49 %) demonstrated low IgG avidity.

**Conclusion:**

Recent travel history, age, or nationality were not statistically significant factors in determining seropositivity. The results of this study highlight the widespread presence in waste workers, where one in five was seropositive for *T. gondii* with no significant differences in both cities. Our findings emphasise the need for implementing preventive measures within a One Health framework to control the spread of toxoplasmosis in the environment, the food industry and the community.

## 1 Introduction

Toxoplasmosis is a significant global health concern, with prevalence rates ranging from 10 to 90 % worldwide (Bisetegn et al., 2023). It is a neglected parasitic disease caused by Apicomplexa intracellular protozoa, *Toxoplasma gondii*. Felines, particularly wild and domestic cats, are the definitive hosts. Oocysts released with the definitive host's faeces can contaminate soil, food, and water. The parasite can be transmitted to humans indirectly from the ingestion of infective oocysts in these sources or by direct contact with infected cats' excreta (Bisetegn et al., 2023; Milne et al., 2020).

Infection with *T. gondii* can cause serious health issues, particularly in immunocompromised patients, pregnant women and neonates (Milne et al., 2020). In immunocompetent individuals, infections are often asymptomatic. However, the parasite can remain in tissues in an inactive phase, known as bradyzoites within cysts, and can reactivate if patients become immunocompromised, causing tissue damage and inflammation. In addition, numerous reports have stated the potential impact of latent toxoplasmosis on human neuropsychological behaviour, including immunocompetent individuals (Milne et al., 2020; Martinez et al., 2018).

Toxoplasmosis is often associated with poor-income populations and low sanitary practices (Martinez et al., 2018). Although Kuwait is considered a high-income country, several factors might influence the transmission of the disease. There are no official governmental statistics on the number of stray cats in Kuwait, despite it being published in the grey literature as a major and neglected concern (Alsaeed, 2018). The current abundance of stray cats in residential areas in Kuwait, which is unregulated by the government, would increase the potential risk of environmental contamination. To date, there are no preventive or control measures implemented by the Public Authority of Agriculture Affairs and Fish Sources, the governing body responsible for the control of stray cats and other animals in Kuwait. Infected stray cats extensively release oocysts into the environment at an early-stage post-infection, and these oocysts are highly resistant to many environmental conditions. Thus, they can last up to a year until infecting another host (Shapiro et al., 2019; Hatam-Nahavandi et al., 2021). In addition, the ability of *T. gondii* to infect various hosts increases the chances of contaminating the human food chain. These factors collectively may facilitate the spread of *T. gondii* within Kuwait's community (Milne et al., 2020; Shapiro et al., 2019; Hatam-Nahavandi et al., 2021).

The trend of increased populations of stray cats is evident in the Middle East and the Gulf Cooperation Council (GCC) region, where studies have reported substantial seropositivity rates among stray cats, indicating a growing risk of *T. gondii* transmission (Abdelgadier et al., 2023). A study conducted in 2013 reported that 20 % of stray cats in Kuwait were seropositive for *T. gondii* (Abdou et al., 2013). Additionally, a study in Qatar published in 2016 found that 82 % of stray cats were seropositive for *T. gondii* (Boughattas et al., 2017). Taking into consideration the similarities in the demographic status and living conditions of Qatar and Kuwait emphasises the need to mitigate the risk of toxoplasmosis transmission.

Municipal waste cleaners were chosen as a target population for this study due to their potentially higher risk of exposure to stray cats and their excreta. Other factors were also considered, including their lower socioeconomic status, income and educational level compared to the general population of Kuwait.

The objective of this study was to estimate the seroprevalence of *T. gondii* among waste collectors across two different governorates in Kuwait – Kuwait City and Jahra. The seropositivity of *T. gondii* antibodies (IgM and IgG) was measured using the Elecsys *T. gondii* (Roche Diagnostics). Additionally, the determination of *T. gondii* IgG avidity was used as a verified technique to distinguish recent from previous infections in human sera (Murat et al., 2012).

## 2 Material and methods

### 2.1 Study design and study area description

A cross-sectional study was conducted in February 2023 to estimate the seroprevalence of *Toxoplasma gondii* among waste workers. Data collection involved administering a structured questionnaire to participants prior to collecting their blood samples for *T. gondii* antibodies (IgM, IgG and IgG avidity).

**Fig. 1:**
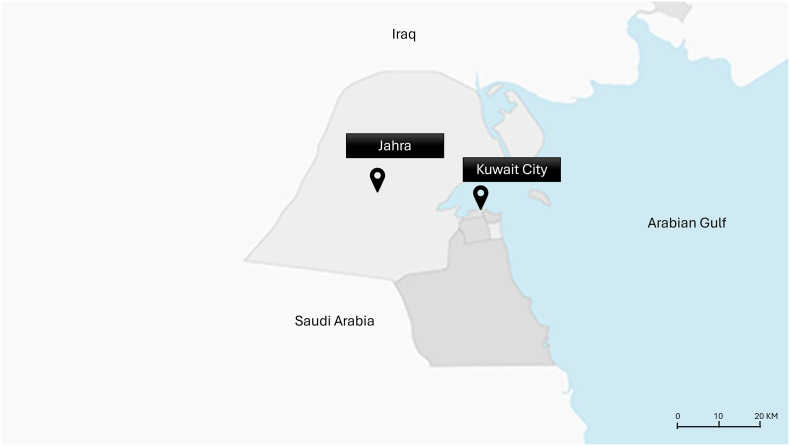
The map of Kuwait illustrates the two studied governorates, Kuwait City and Jahra.

Two waste workers' residential sites were visited to collect the samples: Sabhan and Sulaibiya, representing Kuwait City and Jahra, respectively. During the time of the study, all waste workers in Kuwait were male and lived in their government-designated residential complexes within each city. 201 waste collectors were randomly selected from two Municipal waste cleaners' residential sites, covering various areas in two governorates of the state of Kuwait: Kuwait City and Jahra. The research team visited the two sites on separate dates to collect blood samples and administer questionnaires. These two cities were selected for the study due to their socio-economic differences. Kuwait City, the Capital of Kuwait, is a highly urbanised area, serving as the hub for Government administrative services and commercial activities. In contrast to Jahra, it is located 30 km west of Kuwait City, as shown in Fig. 1, and is known for its agricultural and less affluent identity (Environment Public Authority (EPA), 2019).

For the sampling techniques, residential barracks housing the waste workers in each city were randomly selected at each site, and all workers residing in every third barrack were included in the study. As the number of waste workers in Jahra was higher than in Kuwait City, according to the National Cleaning Company, which operates in collaboration with the Kuwait Municipality, a larger sample was taken from that city. Inclusion criteria were active employment as waste workers at the National Cleaning Company and residency in the target areas for at least a year prior to the study. Participants who did not meet these criteria were excluded.

### 2.2 Sample size

There is a dearth of recent studies on the prevalence of *T. gondii* in Kuwait and other Gulf Council countries, with no studies on the selected population for this study and on the general population. One recent study found that 12.5 % of pregnant women in Kuwait were IgG seropositive for *T. gondii* (Al-Shammari and Iqbal, 2021). This estimate of 12.5 % was used in calculating the sample size needed for this study at a 95 % confidence level with a margin of error of 5 % with an estimated population of approximately 5000 waste workers (including temporary workers) for the State of Kuwait. This estimates a minimum sample size of 163 workers for the parameters mentioned above from both cities.

### 2.3 Sample collection

Two registered nursing staff members collected 201 participants' venous blood samples (5 ml) at both sites. The sample size was increased beyond the estimated number to enhance the precision of the study's findings. Prior to the visit, two meetings were held with the nursing staff to confirm that they followed the same study protocol. The collected samples were transported to the Parasitology laboratory (at the College of Allied Health Sciences, Kuwait University) in temperature-controlled containers. The serum was separated by centrifugation and aliquoted into microtubes for storage at -20 °C for subsequent serological testing.

### 2.4 Demographic data

Key demographic details were obtained from all participants at the time of blood collection via a short questionnaire in four languages (Arabic, English, Hindi, Bengali). The questionnaire was validated at Kuwait University in a focus group with native speakers of the four languages in December 2022. The questionnaire included three questions on age, nationality, and history of recent travel (defined as less than six months before taking the blood sample) to assess for potential confounding factors. As all subjects were male workers, no questions regarding sex were included. Answers were written by the participants and collected before withdrawing the blood sample.

### 2.5 Chemiluminescence immunoassay

#### 2.5.1 IgM and IgG serology test

The prevalence of *T. gondii* antibodies (IgM and IgG) in the serum of waste collectors was measured using Elecsys IgM and IgG antibodies kits (Roche Diagnostics GmbH, Mannheim, Germany). Neat serum samples were tested with a chemiluminescence immunoassay automated analyser (Cobas e411, Roche Diagnostics) at Yacoub Behbehani Specialised Laboratory Centre (Virology Laboratory at Alsabah Medical Area, Kuwait), conducted in compliance with the kit's technical specifications. Antibody concentrations were measured and expressed in international units per millilitre (IU/ml). The results were interpreted according to the kit manufacturer's instructions, as shown in Table 1.

**Table 1:** Cutoff index values of Elecsys Toxoplasma gondii IgM and IgG antibodies according to Roche Diagnostics kit instructions.

*T. gondii* antibody	Negative	Indetermined	Positive
IgM	< 0.8 cutoff index	(≥ 0.8 - < 1) cutoff index	≥ 1 cutoff index
IgG	< 1 IU/ml	(≥ 1 - < 3) IU/ml	≥ 3 IU/ml

#### 2.5.2 IgG avidity test

All IgG seropositive samples were further tested for *T. gondii* IgG avidity (Elecsys, Roche Diagnostics) using the automated Cobas e411 analyser. Samples were first diluted with universal and *Toxo* avidity diluents, both of which were provided with the kit, before undergoing the automation process as instructed. The percentages of IgG avidity were calculated for the tested serum samples according to the kit manufacturer's recommendations, and the results were interpreted as shown in Table 2.

**Table 2:** Cutoff index values (percentages) of Elecsys Toxoplasma gondii IgG Avidity according to Roche Diagnostics kit instructions.

Percentages of avidity	Results interpretation
< 70 %	Low avidity
70–79 %	Indeterminate
≥ 80 %	High avidity

#### 2.5.3 *Toxoplasma* IgG and IgM titre determination

Serial dilutions were manually performed on all seropositive samples using the dilution buffer provided with the Elecsys *Toxo* IgG kit (Elecsys, Roche Diagnostics). The dilution scheme is detailed in Table 3. All diluted samples were analysed using Cobas analyser (Cobas e411, Roche Diagnostics), alongside calibrators and controls. Antibody concentrations were measured and expressed in international units per millilitre (IU/ml).

**Table 3:** Serial dilutions of Toxoplasma IgG seropositive samples.

Dilution Factor	Serum Volume (μL)	Diluent Volume (μL)	Final Volume (μL)
1:2	100	100	200
1:4	100	100	200
1:8	100	100	200
1:16	100	100	200
1:32	100	100	200
1:64	100	100	200
1:128	100	100	200
1:256	100	100	200
1:512	100	100	200
1:1024	100	100	200

### 2.6 Statistical data analysis

Statistical data analysis in this study was done using GraphPad Prism, version 9.5.1. Chi-square was used for all the statistical analyses to determine the relationship between two categorical variables. Statistical significance was considered at *P* ≤ 0.05.

### 2.7 Ethical consideration

Informed consent was obtained from all participant workers in four languages (Arabic, English, Hindi and Bengali), and participation was voluntary. Four and six workers refused to participate in the study in Kuwait City and Jahra, respectively. This project received ethical approval from Kuwait University (Project number ZN02/21) and the Ministry of Health Ethical Committee in Kuwait (Project number 1708/2021).

## 3 Results

### 3.1 Demographic data

**Fig. 2:**
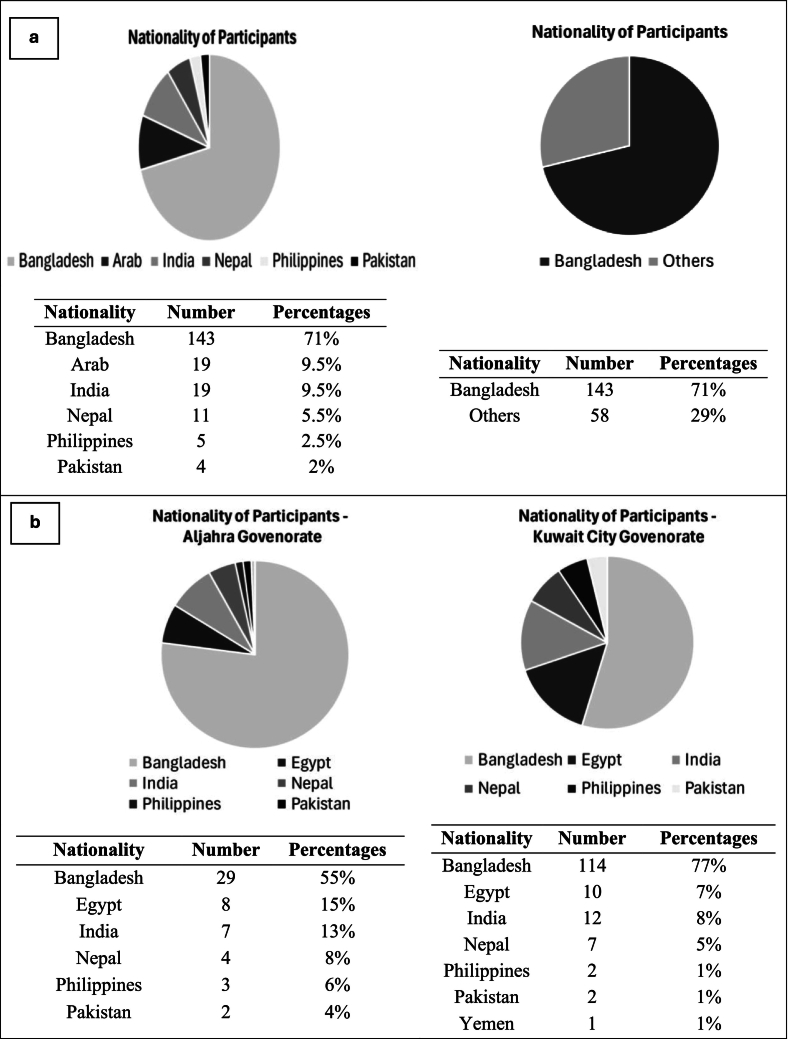
Nationality percentages of all participants ^a^, in Kuwait City and in Jahra governorates in Kuwait ^b^.

A total of 201 male waste collectors participated in the study, with 53 working in areas under Kuwait City governorate and 148 under Jahra governorate. All workers were non-nationals and immigrated from different low-income developing countries, including Bangladesh, Egypt, India, Nepal, Philippines, Pakistan, and Yemen. The majority of workers were Bangladeshis. Fig. 2 shows the nationalities of all participants at both municipal waste collection sites: at Kuwait City and Jahra.

**Fig. 3:**
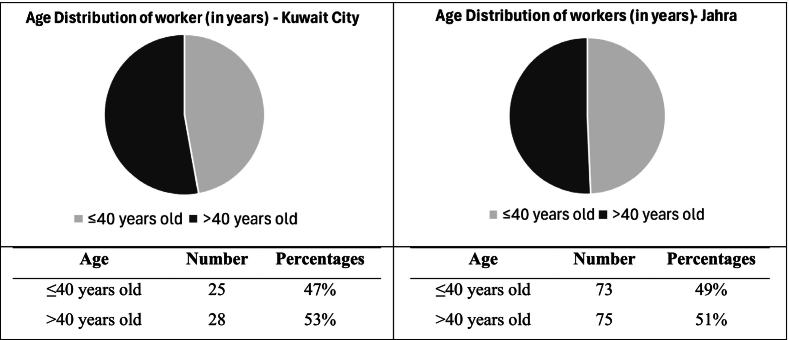
Age Distribution of workers in Kuwait City and Jahra: A comparison between two age groups (40 years and younger vs. older than 40 years).

Workers' ages ranged between 19 and 70 in both governorates. Workers older than 40 comprised 51 % of all participants in both cities. Fig. 3 shows a similar trend in age distribution in both governorates, with both age groups being almost equal in number.

**Fig. 4:**
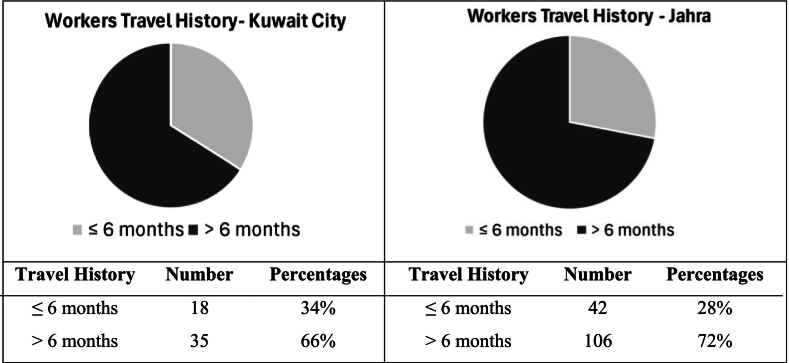
Travel history of waste workers in Kuwait City and Jahra, expressed as percentages: Workers with a travel history of six months or less indicated as recent travel.

The history of recent international travel was recorded for all participants. Workers who had travelled within six months or less at the time of blood collection were considered recent international travellers. As shown in Fig. 4, most workers in the study did not have a recent travel history (70 %): 66 % and 72 % of waste collectors had not travelled internationally in the past six months, in Kuwait City and Jahra, respectively.

### 3.2 IgG and IgM seroprevalence

The seroprevalence of *Toxoplasma gondii* IgG antibodies in the 201 tested samples from both cities was 21 % (43 workers). Among the highest IgG seropositive samples were Bangladeshis (63 %), followed by Arabs (18.6 %), Indians (9.3 %) and Nepalis and Filipinos (each at 4.6 %). Interestingly, only two workers (1 %) tested positive for *T. gondii* IgM antibodies. One Filipino worker in Jahra tested positive for IgM but negative for IgG antibodies. The other worker, an Egyptian working in Kuwait City region, tested positive for both IgM and IgG antibodies.

**Fig. 5:**
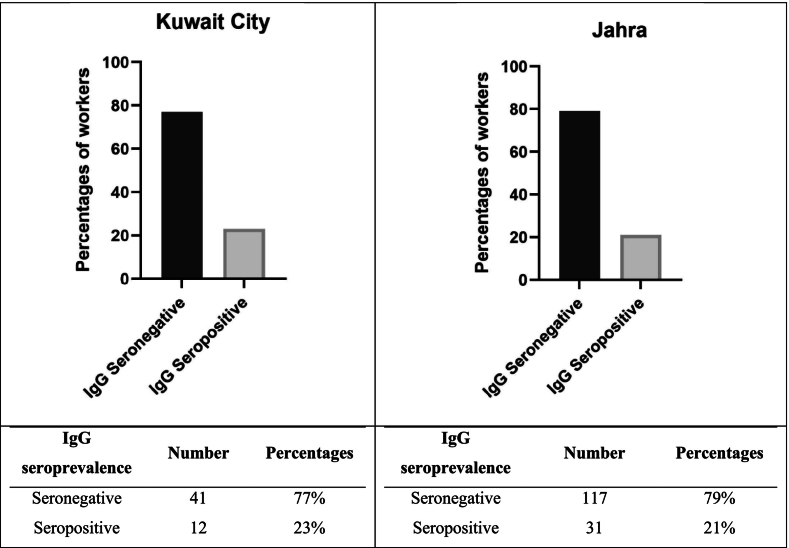
The Seroprevalence of *Toxoplasma gondii* IgG antibodies among waste workers in Kuwait City and Jahra, expressed as percentages.

Out of 53 tested workers in Kuwait City, 12 (23 %) were IgG-positive. Most seropositive workers in this governorate were from Arab countries (42 %), followed by Bangladesh (25 %), the Philippines (17 %), Nepal, and India (each at 8 %). In Jahra governorate, 148 workers were tested, and 31 (21 %) showed reactive IgG antibodies in their sera. The majority were from Bangladesh (77 %), followed by Egypt and India (each at 10 %) and Nepal (3 %). There was no statistically significant difference in the number of workers with IgG positivity when comparing the two governorates (Chi-square test, χ^2^ = 0.12, df = 1, *p* = 0.8), as seen in Fig. 5. Therefore, in further analysis, the data was analysed by combining the seropositive cases from both cities.

Overall, Bangladeshis represented the highest nationality among workers who tested positive for IgG. However, Bangladeshis were not significantly more likely to test positive compared to all other nationalities combined (Chi-square test, χ^2^ = 2.3, df = 1, *p* = 0.2). Age was not found to be a significant factor in seroprevalence. No statistically significant difference was observed in terms of age between participants who tested positive for IgG and those who tested negative (Chi-square test, χ^2^ = 0.2, df = 1, *p* = 0.7) between those below 40 and above 40 years in both cities. Moreover, the proportion of workers with seropositive IgG antibodies was similar to those aged 40 years or younger (51 %) and those older than 40 (49 %).

**Fig. 6:**
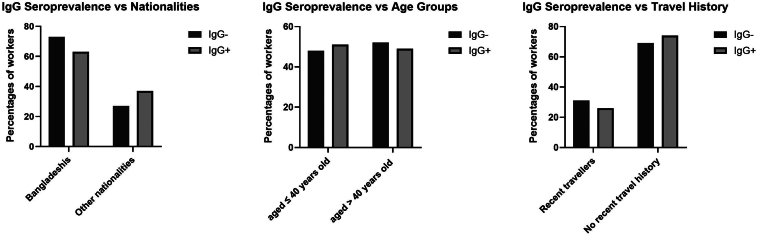
The seroprevalence of Toxoplasma gondii IgG positivity in percentages among waste workers in relation to their nationalities, age, and recent travel history.

An interesting finding is that waste workers who had not travelled outside of Kuwait in the past six months prior to blood collection were more likely to test positive for IgG antibodies than those who had travelled recently. Similarly, no statistically significant difference was found in the number of workers testing positive for IgG in recently travelled workers compared to those with no recent travel history (Chi-square test, χ^2^ = 0.6, df = 1, *p* = 0.4). The two seropositive workers for IgM antibodies denied any recent travel history. *T. gondii* IgG seropositive data concerning the three mentioned factors are presented in Fig. 6.

### 3.3 IgG avidity

To confirm the onset of infection within the studied population, all 43 samples from both cities that seroconverted to a positive reaction against IgG antibodies, regardless of IgM status, were tested for IgG avidity. After determining the avidity percentages as indicated in the manufacturer's kit, the results were analysed. Among the 43 IgG-seropositive waste workers, 21 (49 %) had low IgG avidity, 11 (25.5 %) had high IgG avidity, and 11 (25.5 %) fell within the grey zone. Markedly, these samples were seronegative for IgM antibodies, except for one. This worker, originally from Egypt, tested positive for both antibodies and had high IgG avidity.

The percentages obtained for IgG avidity were compared to demographic variables: nationality, age, and recent travel history of workers. Table 4 presents data on the IgG avidity of workers across various demographic factors. Workers with high and low avidity percentages were almost equally distributed between the two age groups. Participants with low IgG avidity were more likely to have stayed in Kuwait without a recent travel history. Additionally, Bangladeshi workers had lower IgG avidity when compared with other nationalities. However, none of these factors were statistically significant compared to workers with high IgG avidity values.

**Table 4:** *Toxoplasma gondii* IgG Avidity data of waste workers across three different factors: age, recent travel history, and nationality.

	High IgG avidity	Grey-Zone	Low IgG avidity
Total	11	11	21
Age group ≤ 40	6 (55 %)	5 (45 %)	11 (52 %)
Age group > 40	5 (45 %)	6 (55 %)	10 (48 %)
Recent travel history	1 (9 %)	2 (18 %)	7 (33 %)
No recent travel	10 (91 %)	9 (82 %)	14 (67 %)
Bangladeshis	7 (64 %)	6 (55 %)	14 (67 %)
others	4 (36 %)	5 (45 %)	7 (33 %)

### 3.4 Antibody titre determination

A total of 44 seropositive samples were subjected to serial dilutions at the following endpoints: 1:2, 1:4, 1:8, 1:16, 1:32, 1:64, 1:128, 1:256, 1:512 and 1:1024. Of these, 43 samples were tested for reactivity to *T. gondii* IgG antibodies; one sample was reactive for both IgG and IgM, and one sample was tested for IgM antibodies alone, each at different dilution endpoints. Reactivity at each dilution was classified as: reactive, indeterminate or non-reactive, according to the interpretation criteria outlined in the Elecsys *Toxo* IgG and IgM assay protocols (Elecsys, Roche Diagnostics). The full dilution reactivity profiles are summarised in Table 5.

**Table 5:**
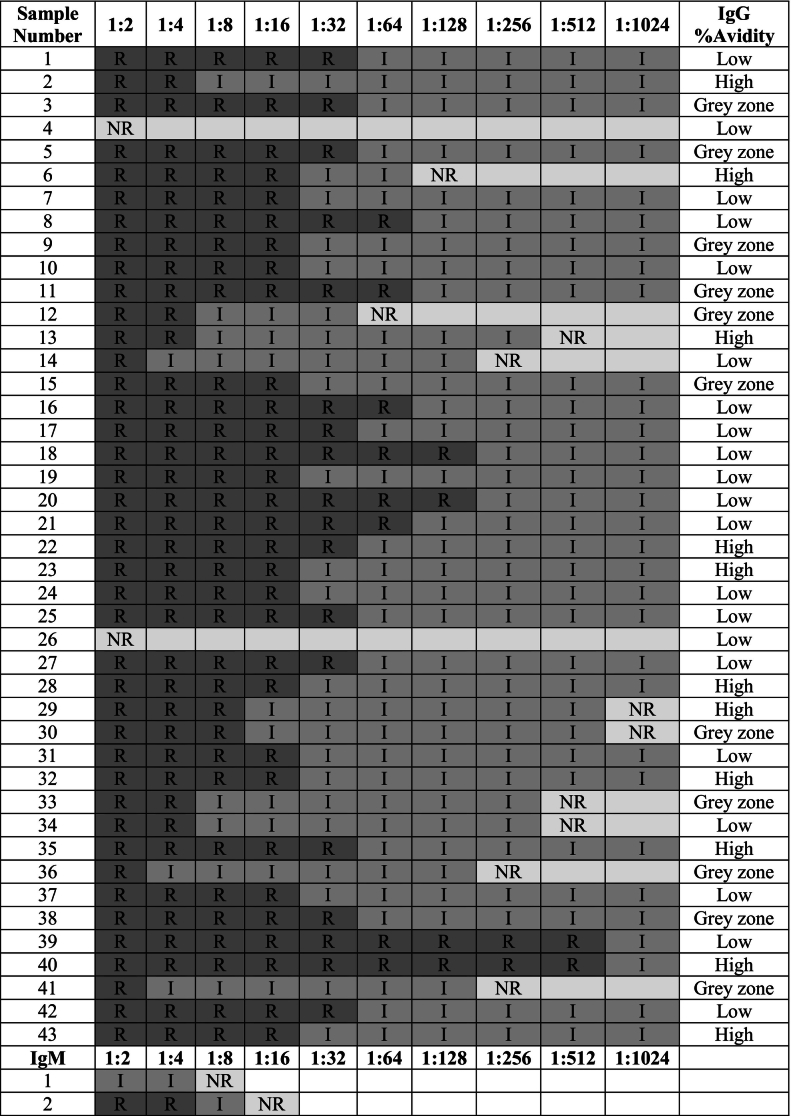
Reactivity of Toxoplasma Seropositive Samples Across Serial Dilutions. Reactivity is classified as: reactive (R, dark grey), indeterminate (I, medium grey), and non-reactive (NR, light grey). The table also presents antibody titration results in relation to corresponding IgG avidity percentages, allowing comparison between dilution profiles and avidity indices.

Among the 43 IgG seropositive samples, two were found to be non-reactive at the lowest dilution (1:2). The remaining samples exhibited varying levels of reactivity across the dilution series, with most retaining reactivity at mid-range dilutions. When analysed in conjugation with IgG avidity data, most samples (11 out of 19) with low avidity remained reactive at intermediate dilutions (particularly at 1:16 and 1:32) before transitioning to indeterminate or non-reactive at higher dilutions. Notably, 6 out of 19 low avidity samples continued to show reactivity at higher dilutions (1:64 and 1:128). Similarly, samples with high IgG avidity also displayed variable dilution profiles. While 5 out of 11 remained reactive at intermediate dilutions (1:16), an equal subset of three samples lost reactivity at lower dilutions (1:4–1:8) or maintained reactivity at higher dilutions (up to 1:32 and even 1:512). Samples with grey zone avidity did not exhibit a consistent pattern and instead showed scattered endpoint reactivity across the serial dilutions.

Regarding the IgM-positive samples, the sample that tested positive for both IgG and IgM antibodies was retested following dilutions. This sample showed indeterminate IgM reactivity at 1:2 dilution and lost reactivity entirely at 1:8. The second IgM-positive sample, which was IgG negative, demonstrated reactivity at the lowest dilutions, became indeterminate at 1:8, and turned non-reactive at a 1:16 dilution.

## 4 Discussion

This study investigated the seroprevalence of *Toxoplasma gondii* antibodies in waste collectors in Kuwait in two different governorates. This target population was chosen due to their potentially higher occupational health risk of exposure to stray cats and their fluids and excreta, a common route of transmission.

A total of 1 % and 21 % of waste workers had *T. gondii* IgM and IgG antibodies in their sera, respectively. Typically, IgM antibodies would appear within the first week post-infection and afterwards decline fast, while IgG antibodies would follow the IgM appearance from one to two weeks (Ybañez et al., 2020). Hence, IgM is usually used as an early marker for recent *T. gondii* infections, and IgG antibodies would indicate past infections or gained immunity. However, in some cases, IgM antibodies can persist for months and even years post-infection (Ybañez et al., 2020). Therefore, the seroprevalence of IgM and IgG antibodies in a single serum sample would not necessarily differentiate recent from past infections. Using the IgG avidity test is a validated method to determine the onset of infection (Teimouri et al., 2020; Candolfi et al., 2007). The principle of IgG avidity testing is based on measuring the binding strength of *T. gondii* IgG antibodies, referred to as the IgG avidity index (AI). This index (in percentages) reflects the binding affinity of antibodies, which would increase gradually over weeks or even months following exposure to the parasite's antigens (Teimouri et al., 2020; Garnaud et al., 2020). This study used the Elecsys *Toxo* IgG avidity assay (Roche Diagnostics, GmbH, Germany), which utilised urea as a protein digestant agent to disrupt the weak binding of low-affinity antibodies to the antigens presented. As a result, a low IgG AI signifies weaker antibody-antigen interaction, whereas a high AI reflects stronger affinity binding. This distinction is instrumental in differentiating acute from chronic infections (Teimouri et al., 2020).

It is worth noting that the interpretation of the IgG avidity differs based on the commercial avidity assay used in the experiment. We have further tested all seropositive IgG samples for IgG avidity, regardless of their IgM status. This approach follows the recommendations from several studies to ensure early infections are not missed in the absence of IgM antibodies (Teimouri et al., 2020; Fricker-Hidalgo et al., 2013; Ikuta et al., 2023).

As 26 % of IgG seropositive workers had high avidity, this would exclude the possibility of recent infections within the last 4 months and may suggest that those workers had *T. gondii* infections in the past 9 months (Murat et al., 2012; Teimouri et al., 2020; Garnaud et al., 2020). Interestingly, 49 % of workers demonstrated positive IgG antibodies and low IgG avidity, with no IgM in their sera. In certain cases of recent infections, as IgM antibodies begin to decline in serum, IgG levels rise, initially exhibiting low avidity that increases over time. This pattern has been documented during the early phases of *T. gondii* infections, as well as in Cytomegalovirus (CMV) infections (Villard et al., 2016; Laboudi and Sadak, 2017). Nevertheless, low avidity can persist for up to a year (Holec-Gąsior and Sołowińska, 2022).

The antibody titration results provided additional insight into the strength and persistence of the IgG response in seropositive samples. Notably, 95 % of these samples maintained reactivity at intermediate to high dilutions, mostly at 1:16 and 1:32, with a few reaching up to 1:512, indicating moderate to high antibody concentrations. This supports the initial interpretation that these were true positive results. However, no clear pattern emerged when our results were compared to IgG avidity data. Samples with either high or low avidity generally maintained reactivity at intermediate dilutions and did not test negative at higher dilutions (up to 1:1024). The experiment was halted before endpoint reactivity could be reached in most cases due to resource limitations. Similar findings have been reported in other studies, where high *T. gondii* antibody titres were associated with low IgG avidity indices and positive PCR results, indicative of recent infection (Teimouri et al., 2020; Berredjem et al., 2017). In contrast, Benner et al. demonstrated that high avidity antibodies to severe acute respiratory syndrome coronavirus2 (SARS CoV2) were linked to elevated antibody titres and consistent with past infections (Benner et al., 2020). These observations, along with our data, suggest that antibody titre levels do not reliably correlate with the IgG avidity indices. This discrepancy limits their utility in effectively determining the timing of infection.

Low avidity values (defined as less than 19 % AI value using the Elecsys avidity assay) can confirm very recent infections of three months and less (Murat et al., 2012; Teimouri et al., 2020). In our study, one worker in Kuwait City and three in Jahra demonstrated low avidity percentages below 19 %. However, interpreting avidity in low-titre samples requires caution. Two of three samples (comprising 5 % of all seropositive samples) initially appeared reactive but showed no reactivity after the first titration. These cases may present false positives, extremely weak immune responses, or very recently acquired infection and therefore require further evaluation (Holec-Gąsior and Sołowińska, 2022).

Only one worker demonstrated seropositivity for both IgM and IgG antibodies yet exhibited a high avidity percentage. Upon antibody titration, the IgM response was indeterminate even at the lowest dilution and became non-reactive at a relatively low dilution level. This IgM seropositivity may represent a false positive result or a possible case of reactivation, both of which warrant further investigation for confirmation (Ybañez et al., 2020).

Approximately one out of four workers from both cities had IgG avidity within the grey zone, making interpretation difficult. For these samples, recent infections cannot be ruled out without follow-up testing or confirmation using alternative serological assays (Murat et al., 2012; Garnaud et al., 2020). The lack of a consistent dilution pattern among grey zone samples suggests a borderline or unstable antibody response, potentially reflecting variability in antibody titre level, immune status, or assay sensitivity. Unfortunately, follow-up testing was not feasible due to the anonymous and random nature of blood sample collection, which prevented further analysis or evaluation of antibody titre changes over time.

Serological tests have been the primary method for evaluating toxoplasmosis in humans in many epidemiological studies, detecting single or multiple antibodies (IgM, IgG and IgG avidity) against *T. gondii* across diverse population groups (Bisetegn et al., 2023; Al-Shammari and Iqbal, 2021; Ybañez et al., 2020; Candolfi et al., 2007; Laboudi and Sadak, 2017; Mohammed et al., 2024). Techniques like agglutination tests, indirect fluorescent antibody tests, and enzyme-linked immunosorbent assay (ELISA) are effective for antibody detection (Deshmukh et al., 2021). Cross-reactivity with other protozoa, such as *Hammondia hammondi* and *Neospora caninum,* cannot be excluded when conducting serological testing. However, it is less frequently observed when using recombinant antigens, and these species are not yet known to cause infections in humans (Gondim et al., 2017). While chemiluminescence immunoassay and ELISA demonstrate comparable sensitivity, the former offers the advantage of being less labour intensive and faster to perform (Murat et al., 2012; Holec-Gąsior et al., 2018). The Elecsys *Toxo* IgG assay demonstrates a sensitivity of 99.5–100 % and a specificity of 88–99.8 %. In comparison, the IgM assay has a sensitivity of 91–96 % and a specificity of 98.5–99.8 % (Meylan et al., 2015). The percentage of false positives for IgG using the Elecsys serological assay is estimated to be relatively low, at 3.6 % (Villard et al., 2016). Serological diagnosis of toxoplasmosis can be challenging to interpret due to the reasons mentioned in this study. In such cases, antigen detection methods or molecular testing can be used to confirm recent or current infections, especially among at-risk groups. However, this is generally not done due to high economic costs (Fricker-Hidalgo et al., 2013), and was not conducted in this study due to funding constraints. Nevertheless, serological avidity testing is as effective as polymerase chain reaction (PCR) in identifying early infections for toxoplasmosis. However, in cases of grey zone/intermediate or even low AI values, which may persist, PCR proves to be more reliable (Holec-Gąsior and Sołowińska, 2022; Berredjem et al., 2017; Bahadori et al., 2019).

Although the majority of seropositive waste workers had not travelled outside Kuwait within the past six months, recent travel history was not a statistically significant factor in determining seropositivity. It is worth noting that travelling every year may not be a viable option for many waste workers due to their low socioeconomic status and the financial burden of air travel to their country of origin. The possibility that these workers were exposed to the parasite while residing in Kuwait cannot be ruled out and requires further research. This suggests the possibility that toxoplasmosis might be endemic in Kuwait, and the source of transmission to these workers and other at-risk populations should be further investigated. It should be noted that a larger sample size would have strengthened the findings of this study. However, due to time and funding constraints, this was beyond the scope of this research.

Toxoplasmosis is well documented globally, with extensive research emphasising its importance, particularly among high-risk groups. Certain occupations, including those involving exposure to animals or raw meat, have been identified as high-risk groups for toxoplasmosis (Mohammed et al., 2024; Hejazi et al., 2023; Amiri et al., 2024). A systemic review and meta-analysis showed global *T. gondii* infection prevalence rates as follows: 54 % of non-livestock animal workers, 47 % of livestock animals, 44 % of slaughterhouse workers, and 27 % of veterinary workers were seropositive for *T. gondii* antibodies. Income status was considered a contributing risk factor (Mohammed et al., 2024). In Iran, 53 % of butchers in Tabriz tested positive for *Toxoplasma* IgG antibodies compared to 32 % in a control group (Amiri et al., 2024). Similarly, a study in Isfhan found higher seropositivity rates among slaughterhouse, livestock, farms, and veterinary workers (46 %) compared to the control group (31.4 %) (Hejazi et al., 2023). As this study was designed as a prevalence study rather than a case-control study, future research can benefit from providing a control group in order to assess the differences between at-risk populations and the general population. Unfortunately, only a few published studies acknowledged the spread of the disease in the Middle East region, especially the Gulf Cooperation Council (GCC) countries, where previous studies primarily focused on pregnant women (Rabaan et al., 2023; Rostami et al., 2020). This is the first study to estimate the prevalence of *T. gondii* in the context of occupational exposure hazards (such as waste workers) in Kuwait and the Middle East region. Waste workers in Kuwait come from low-income countries, such as Bangladesh, Egypt, Nepal, and India. This is not unique to this particular occupation in Kuwait, as expatriates make up approximately 68 % of the population, according to the Public Authority for Civil Information (PACI), and are mostly from developing countries (The Public Authority for Civil Information (PACI), 2023). Factors like poor socioeconomic status, cultural practices, the abundance of stray cats, and climate conditions can all play crucial roles in the prevalence rate of *T. gondii* infections (Al-Malki, 2021).

Although we have attempted to control for potential confounding factors such as nationality, age, and recent travel history, other confounding factors may exist. It is important to note that all participants lived in similar residential conditions and received the same salary as per government regulations for this occupation. Additionally, the residential areas for waste workers are all regulated by the Government of Kuwait in both cities with similar living conditions. However, confounding factors associated with personal hygiene and environmental conditions, which were beyond the scope of this research, could be addressed in future research.

While the prevalence of toxoplasmosis among pregnant women in Kuwait has been decreasing over the years (Al-Shammari and Iqbal, 2021), increasing epidemiological surveillance studies at a community level would help in reducing serious complications of the disease among high-risk populations, including the elderly and immunocompromised patients. These surveillance studies can also be extended to animals and soil testing to evaluate *T. gondii* infection rates. Increasing public awareness and education can promote better hygiene practices in households and the workplace. Additionally, implementing strict governmental policies to regulate food and water safety and animal management, particularly for stray cat populations, would further support prevention efforts.

The results of this study highlight the widespread presence of *T. gondii* infections in waste workers, where one in five was seropositive for *T. gondii*, with no significant differences in both cities. Considering these findings, future studies are needed to determine if similar observations are present in the general population of the State of Kuwait. Implementing preventive measures within a One Health framework is crucial for controlling the spread of toxoplasmosis and other zoonotic parasitic infections, such as *Toxocara* species.

## CRediT authorship contribution statement

**Anfal Yousef:** Writing – review & editing, Writing – original draft, Validation, Resources, Project administration, Methodology, Investigation, Funding acquisition, Formal analysis, Data curation, Conceptualization.

## Funding

This project was funded by the Research Sector of Kuwait University, Kuwait, under grant number ZN0221.

## Declaration of competing interest

The authors declare that they have no known competing financial interests or personal relationships that could have appeared to influence the work reported in this paper.
